# Primary extraskeletal osteosarcoma of the retroperitoneum: a case report

**DOI:** 10.3389/fmed.2026.1921181

**Published:** 2026-10-08

**Authors:** Xiangxiang Ren, Tianhao Xie, Linqian Li, Xiaoshi Jin, Meng Zhang

**Affiliations:** 1 Department of General Surgery, Affiliated Hospital of Hebei University, Baoding, China; 2 Basic Research Key Laboratory of General Surgery for Digital Medicine, Affiliated Hospital of Hebei University, Baoding, China; 3 Department of Dermatology, Affiliated Hospital of Hebei University, Baoding, China

**Keywords:** CDK4, MDM2 amplification, osteoid-producing sarcoma, retroperitoneal extraskeletal osteosarcoma, retroperitoneal sarcoma

## Abstract

Extraskeletal osteosarcoma arising in the retroperitoneum is exceedingly rare, and its osteoid-producing histology closely mimics dedifferentiated liposarcoma. A 33-year-old woman presented with a 1-month history of progressive abdominal distension. Imaging revealed a 15 cm right retroperitoneal mass with punctate calcifications, no macroscopic fat, and no bone attachment. The mass was resected completely *en bloc* with negative microscopic margins (R0). Sections showed a high-grade sarcoma weaving through lace-like osteoid. MDM2 immunohistochemistry was negative and fluorescence *in situ* hybridization (FISH) detected no MDM2 amplification; CDK4 immunostaining and CDK4 FISH were likewise negative. No well-differentiated liposarcoma component was found on gross or microscopic examination, and together these findings ruled out 12q13-15 amplification. The final diagnosis was primary, high-grade extraskeletal osteosarcoma of the retroperitoneum. The patient received adjuvant MAP chemotherapy (methotrexate, doxorubicin, cisplatin) and at 25 months after surgery remains free of recurrence or metastasis; surveillance imaging will continue for at least 10 years. Any osteoid-producing retroperitoneal sarcoma without a well-differentiated liposarcoma component should undergo MDM2 amplification testing, and ideally CDK4 testing, because the two diagnoses are managed differently.

## Introduction

Extraskeletal osteosarcoma is a malignant mesenchymal neoplasm that produces osteoid or bone matrix without attachment to the skeleton. It accounts for less than 1% of soft-tissue sarcomas and roughly 4% of all osteosarcomas (1, 2). The tumor typically arises in the deep soft tissues of the thigh and lower extremity; primary retroperitoneal origin is exceptionally rare (3).

In the retroperitoneum, the principal differential diagnosis is dedifferentiated liposarcoma with osteosarcomatous differentiation, a far more common entity that can produce abundant malignant osteoid and appear histologically indistinguishable from extraskeletal osteosarcoma (4, 5). Dedifferentiated liposarcoma is genetically defined by 12q13–15 amplification involving MDM2 and CDK4; distinguishing these two entities therefore requires molecular testing. Mistaking one for the other alters management: dedifferentiated liposarcoma is treated by complete surgical resection, whereas extraskeletal osteosarcoma may warrant adjuvant chemotherapy by analogy with skeletal osteosarcoma protocols. MDM2 and CDK4 testing should be part of the evaluation of any osteoid-producing retroperitoneal sarcoma.

## Case report

A 33-year-old woman with no significant medical history noticed her abdomen enlarging over 1 month. She had no pain, nausea, or change in bowel habit. Examination revealed a firm, nontender mass occupying the right abdomen. Routine blood tests were normal.

Contrast-enhanced CT showed a 15 cm, heterogeneously enhancing soft-tissue mass in the right retroperitoneum. The mass abutted the cecum and appendix but was separate from the axial skeleton. Punctate and amorphous calcifications suggested osteoid production; central non-enhancing areas were consistent with necrosis. No macroscopic fat was visible within the mass or elsewhere in the retroperitoneal space. This finding makes a sizeable well-differentiated liposarcoma component unlikely, though a tiny one cannot be excluded. MRI was omitted because of clinical circumstances and the CT information in this particular patient. Although MRI would have offered superior soft-tissue contrast and may have better characterized internal tumor heterogeneity, the tumor extent, its relationship to the adjacent vessels, and the absence of bone attachment were already well delineated on CT, and this information was considered adequate for surgical planning in this case. Three-dimensional reconstruction placed the tumor at the ileocecal region, with dominant blood supply from the right colic vessels (Figures 1, 2).

**Figure 1:**
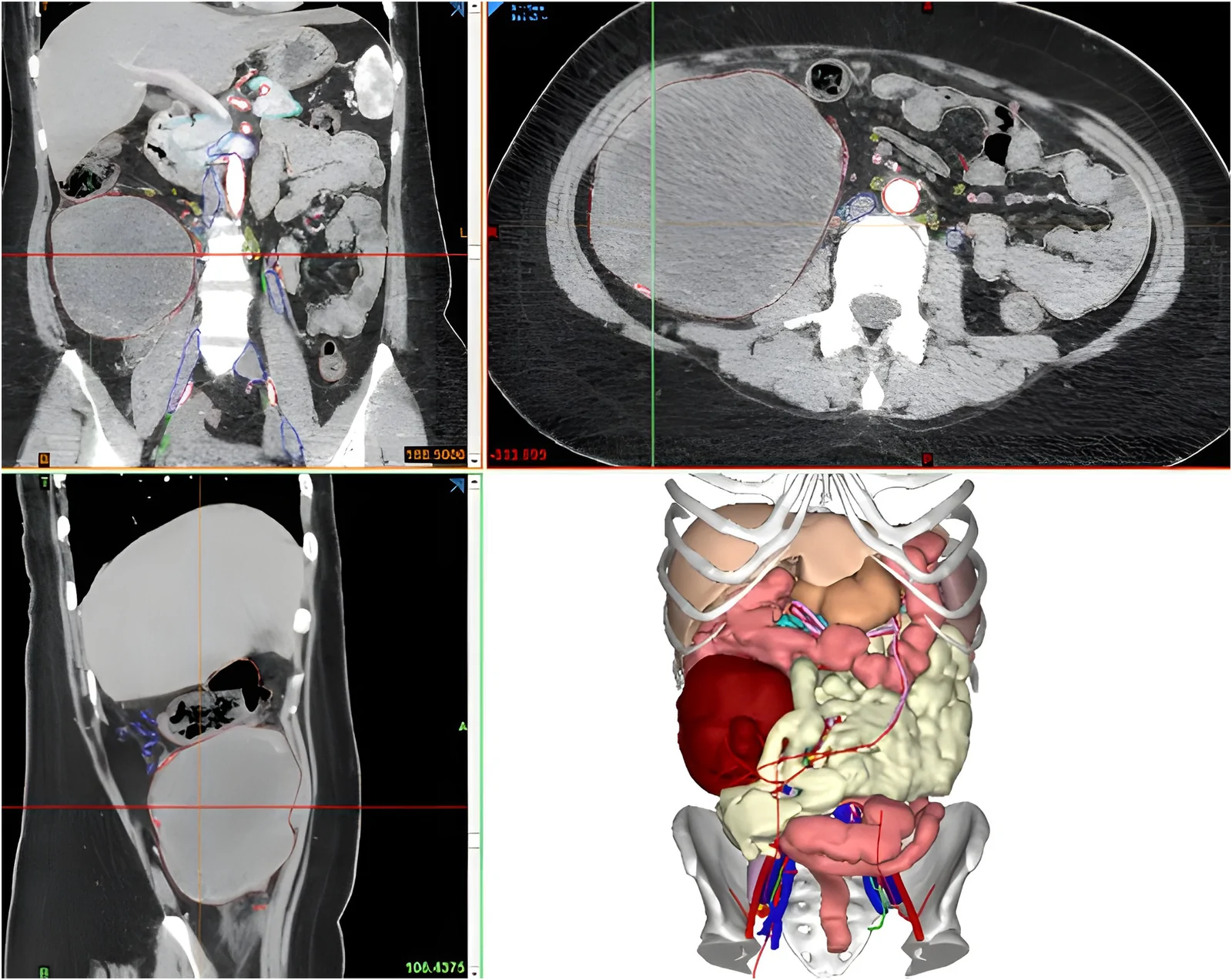
Preoperative contrast-enhanced computed tomography of the retroperitoneal mass. Axial view showing a 15 cm heterogeneously enhancing soft-tissue mass in the right retroperitoneum (arrows) with punctate calcifications and central non-enhancing areas consistent with necrosis. No macroscopic fat is visible within the mass.

**Figure 2:**
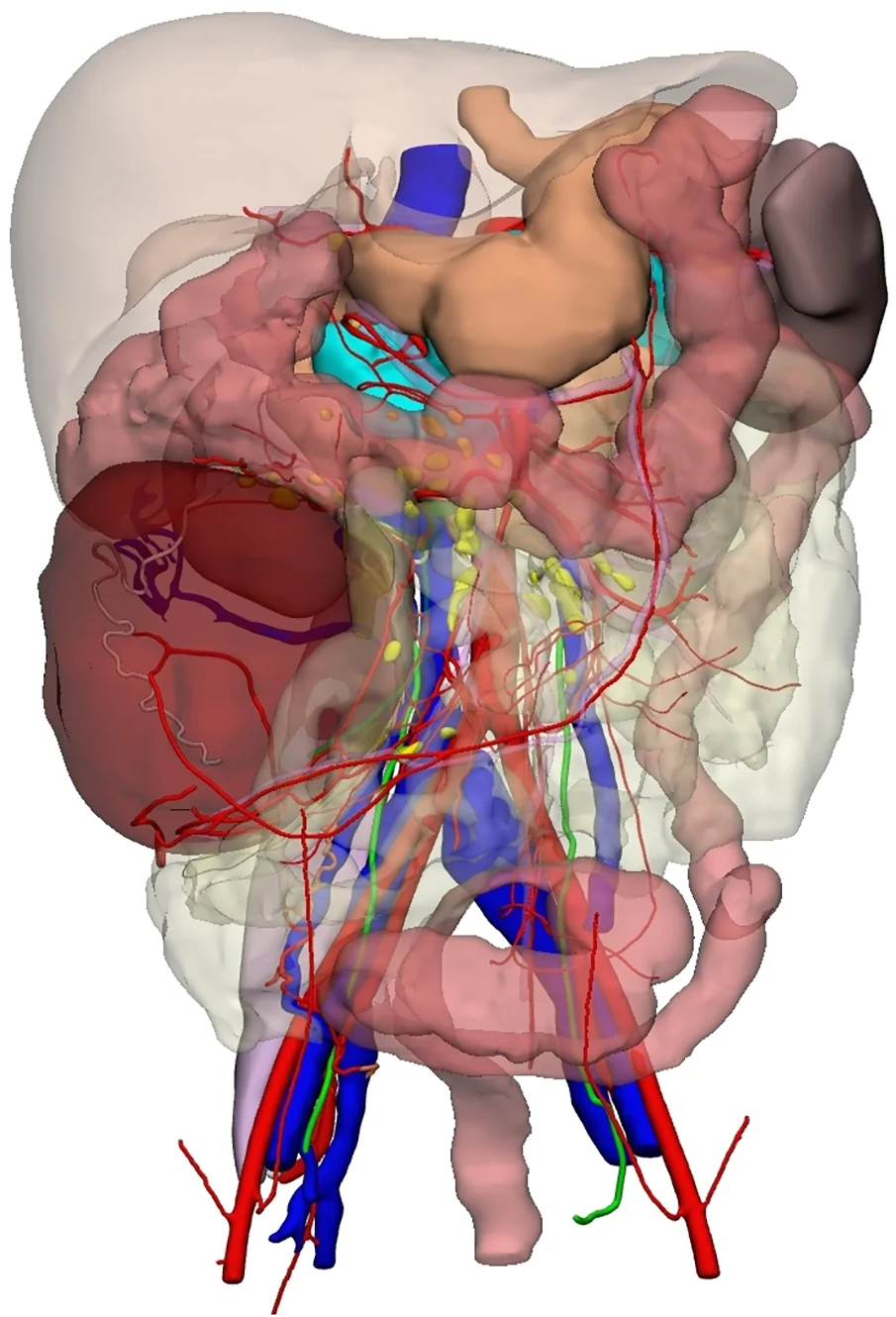
Three-dimensional reconstruction demonstrating the tumor at the ileocecal region, with dominant blood supply from the right colic vessels. The mass is separate from the axial skeleton.

After multidisciplinary discussion, a preoperative core needle biopsy was deliberately omitted. The tumor was large, her distension was progressing rapidly, and imaging suggested resectability. This decision deviated from current guidelines, which recommend biopsy for virtually all retroperitoneal masses (6); it should be read as a cautionary note rather than a model to follow.

En bloc resection of the mass together with the involved ileocecal segment was performed. All resection margins were tumor-free (R0).

## Histopathological findings

The resected tumor measured 15 cm in greatest dimension. The specimen was well-demarcated, unencapsulated, firm, and gray-white, with areas of hemorrhage and necrosis on the cut surface (Figure 3). Gross examination revealed no macroscopic fat component or discrete well-differentiated areas.

**Figure 3:**
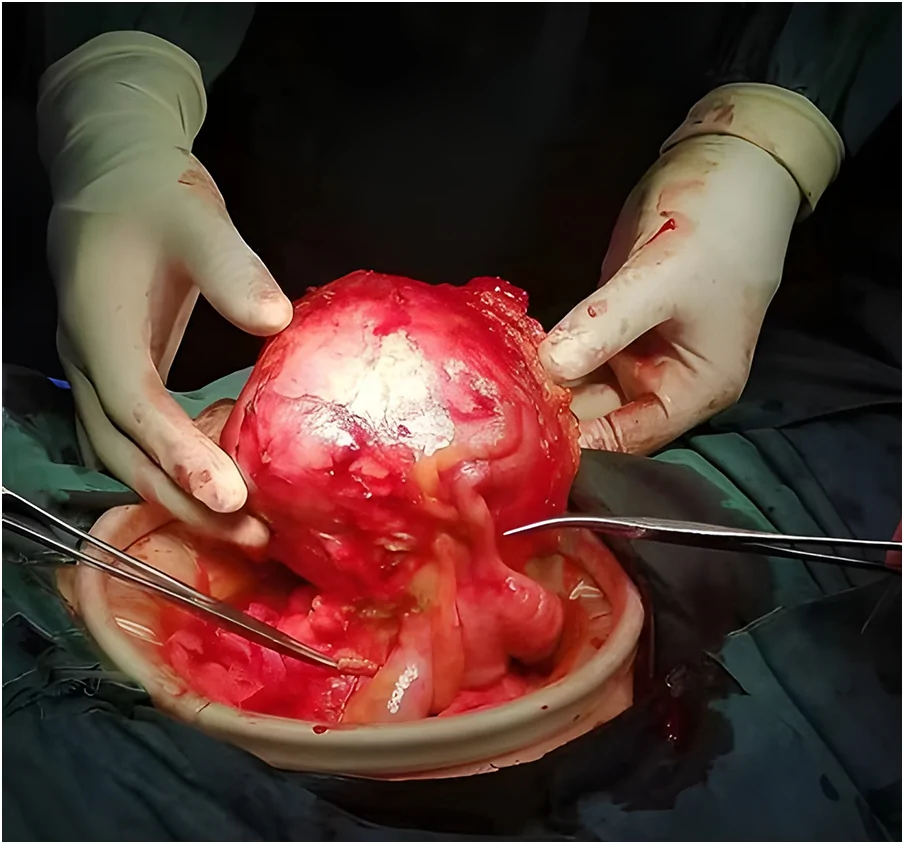
Intraoperative photograph of the resected *en bloc* specimen including the tumor and attached ileocecal segment.

The surgical specimen was serially sectioned at 1 cm intervals. Every nodule, firm area, and region with a color or texture differing from the dominant gray-white sarcoma was sampled, together with generous sections of the tumor–adipose interface. In total, 32 blocks were submitted for histology. No fat was identified on gross or microscopic examination.

Hematoxylin and eosin (H&E) sections showed a densely cellular proliferation of pleomorphic spindle and epithelioid cells with abundant mitoses, woven through a fine, lace-like osteoid matrix (Figure 4). By immunohistochemistry, the tumor cells diffusely expressed SATB2 and vimentin, with focal membranous CD99. They were negative for CD34, smooth muscle actin, desmin, DOG1, pan-cytokeratin, MDM2, and CDK4. The Ki-67 index reached 40%. Given the limited sensitivity and specificity of MDM2 immunohistochemistry (7), FISH was performed and detected no MDM2 amplification. CDK4 lies on the same 12q13–15 amplicon; co-amplification provides additional diagnostic security (8), and CDK4 FISH was also negative. The concordant absence of these amplifications, together with the absence of a well-differentiated liposarcoma component on imaging and exhaustive gross examination, makes dedifferentiated liposarcoma highly improbable. The negative pan-cytokeratin result, along with the absence of CK7 and CK20, supports the mesenchymal nature of the epithelioid component and excludes carcinoma.

**Figure 4:**
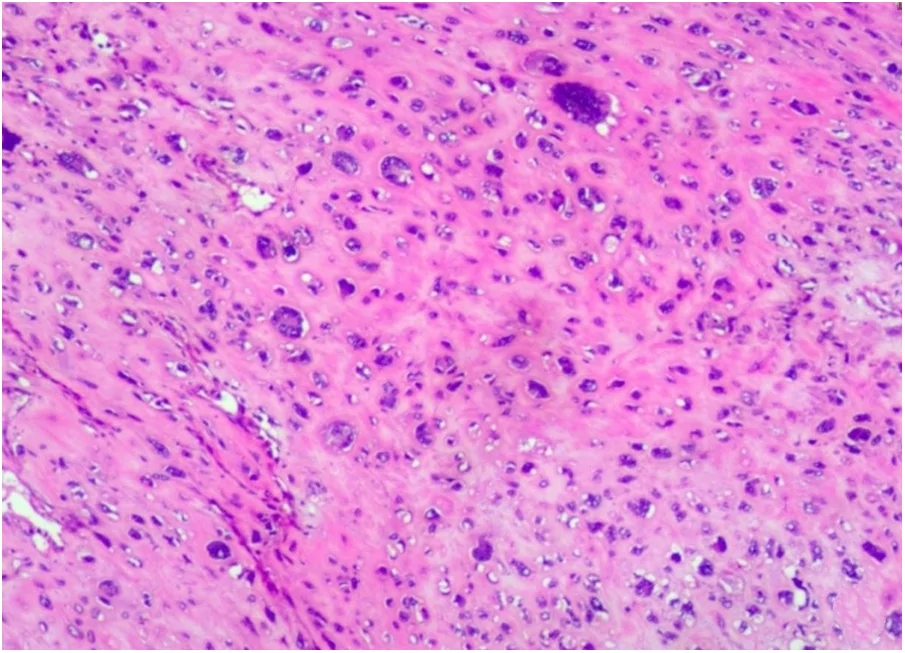
Histopathological and molecular findings. (**A**) In the tumor, densely cellular proliferation of pleomorphic spindle and epithelioid cells with abundant mitotic figures were observed (HE, 100 × ). (**B**) High-power view demonstrating the characteristic lace-like osteoid matrix (HE, 400 × ). (**C**) Immunohistochemistry for SATB2 showing diffuse nuclear positivity.

Other osteoid-producing lesions were considered. Ossifying fibromyxoid tumor typically shows multinodular architecture and co-expresses S100 and desmin, which this tumor did not. Sclerosing epithelioid fibrosarcoma was excluded by the lack of dense stromal sclerosis and unequivocal neoplastic bone; the lace-like osteoid, strong SATB2 positivity, and negative MDM2 and CDK4 findings make that diagnosis unlikely (9). Dedifferentiated chondrosarcoma would be expected to show cartilaginous matrix and S100 positivity, neither of which was present. Mesothelioma with osseous metaplasia was ruled out by cytokeratin negativity and the retroperitoneal origin. Undifferentiated pleomorphic sarcoma (UPS) was excluded by the presence of unequivocal lace-like osteoid production throughout the tumor, which is not a feature of UPS. Malignant peripheral nerve sheath tumor (MPNST) would be expected to show S100 positivity and association with a nerve trunk; neither was present. Synovial sarcoma was ruled out by the absence of cytokeratin and absence of diffuse membranous CD99 positivity. Solitary fibrous tumor typically shows strong, diffuse CD34 positivity, whereas this tumor was CD34-negative. Extraskeletal Ewing sarcoma was also considered because of the epithelioid component and the focal membranous CD99 positivity. This diagnosis was judged unlikely on morphologic and immunohistochemical grounds: the tumor was a pleomorphic spindle-cell and epithelioid sarcoma producing abundant lace-like osteoid throughout, whereas Ewing sarcoma characteristically shows monotonous small round cells with scant cytoplasm and does not produce osteoid, and the CD99 staining was only focal rather than the diffuse, strong membranous pattern characteristic of Ewing sarcoma. EWSR1 rearrangement testing by FISH was not performed. The final diagnosis was primary, high-grade extraskeletal osteosarcoma of the retroperitoneum.

## Treatment and follow-up

Postoperative ^1^⁸F-FDG PET/CT 4 weeks after surgery detected no residual or distant disease. The patient received four cycles of MAP chemotherapy repeated every 21 days: high-dose methotrexate 8–12 g/m^2^ as a 24-hour infusion on Day 1 with leucovorin rescue and serum level monitoring, doxorubicin 25 mg/m^2^ daily on Days 1–3 (total 75 mg/m^2^ per cycle), and cisplatin 90–100 mg/m^2^ on Day 1 with vigorous hydration and forced diuresis. The cumulative doxorubicin dose remained below 450 mg/m^2^.

Serum methotrexate concentrations were measured at 24, 48, and 72 h after the start of infusion. They remained within the expected non-toxic range, with no evidence of delayed excretion. Leucovorin rescue began 24 h after the start of methotrexate at 15 mg/m^2^ every 6 h and continued until the serum methotrexate level fell below 0.1 μmol/L. Toxicity was manageable: grade 2 nausea and grade 3 neutropenia without fever. No cardiac, renal, or neurotoxic events occurred.

Adjuvant chemotherapy was offered because the tumor was high-grade and the patient was young, by analogy with skeletal osteosarcoma protocols (10). A recent propensity score–matched analysis of the Japanese Bone and Soft Tissue Tumor Registry reported that adjuvant chemotherapy in extraskeletal osteosarcoma was associated with a possible improvement in disease-specific survival (hazard ratio 0.66; 95% CI 0.44–0.98; *P* = 0.04) (11). This retrospective signal is encouraging but does not replace randomized trial data. At 25 months after surgery (last follow-up May 2026) the patient remains well, with no local recurrence or pulmonary metastasis on CT. Because osteosarcoma is notorious for late relapses, surveillance imaging will continue for at least 10 years.

## Discussion

A retroperitoneal sarcoma that produces osteoid immediately raises suspicion for dedifferentiated liposarcoma, which is far more common at this site than primary extraskeletal osteosarcoma (4, 5). Only 14 cases of primary retroperitoneal extraskeletal osteosarcoma have been reported to date (Table 1), spanning 1990 to 2024, with a median reported age of 56 years and a male predominance of roughly 2:1. Tumor sizes ranged from 5 to 20 cm. Outcomes were generally poor: among cases with available follow-up, 5 of 11 died of disease, with median survival under 2 years. This extreme rarity of retroperitoneal presentation is consistent with the literature: in the largest series of extraskeletal osteosarcoma, the deep soft tissues of the thigh and lower extremity were the predominant primary sites, with retroperitoneal origin accounting for fewer than 5 percent of cases (3). The male predominance noted in the 14 reported retroperitoneal cases (Table 1) mirrors the demographic pattern observed in extraskeletal osteosarcoma overall. The present case illustrates why molecular testing should not be omitted.

**Table 1:** Reported cases of primary retroperitoneal extraskeletal osteosarcoma.

Author (Year)	Age/ Sex	Size (cm)	Imaging Findings	MDM2/CDK4	IHC/Markers	Treatment	Outcome	Key Features
Present case	33F	15	CT: punctate calcifications, no macroscopic fat, central necrosis; no bone attachment	MDM2 FISH (−) CDK4 FISH (−) MDM2 IHC (−) CDK4 IHC (−)	SATB2+, vimentin+CD99 focal+, CD34−, SMA−, desmin− DOG1−, CK− Ki-67 40%	R0 *en bloc* resection+adjuvant MAP × 4	NED 25 mo	Young female; subtle calcifications; only case with systematic dual MDM2 + CDK4 FISH exclusion
Sapiano et al. (2023) (18)	53M	10	CT: partially cystic ossified RP mass, right iliac fossa; ascites, omental calcifications, mesenteric lymphadenopathy; no bone attachment	NR	Vimentin+(diffuse) CK focal+, SMA focal+, CD68+ Melan-A−, S100−, DOG1− c-kit−, pan-CK−	Unresectable; peritoneal catheter for palliation Chemo: doxorubicin 25 mg/m^2^ d1–3 + cisplatin 50 mg/m^2^ d1–2 q3w	SD after 4 cycles; died shortly after	Most recent RP ESOS case report; full text unavailable for detailed data extraction
Jeo et al. (2024) (4)	50M	NR	CT urography: left RP malignant tumor; intermittent lower-left back pain × 6 mo	NR	Vimentin+, SATB2+, CD99+ CDK4+ (IHC only; FISH ND) S100−, Ki-67 80% CK−, CD117−, CD34−, SMA−, desmin−, EMA−	Laparotomy+multi-visceral resection (distal pancreatectomy, partial gastrectomy, splenectomy, left nephrectomy)	Local recurrence 6 mo DOD ∼20 mo (multiple metastases)	RP origin confirmed by imaging; CDK4 IHC + without FISH confirmation; aggressive multi-organ infiltration
Jaipuria et al. (2014) (14)	38M	18.0 × 18.3 × 17.8	CT: large right RP mass; flank pain+abdominal distension	NR	NR	R0 excision+cisplatin (90 mg/m^2^ d1–2)/doxorubicin (75 mg/m^2^ d3) × 6 q3w + G-CSF	NED 3 yr	Largest RP low-grade ESOS (18 cm); 2nd RP low-grade case; 3-year disease-free survival
Nagpal et al. (2016) (19)	62M	20 × 15	CT: large RP mass infiltrating L3–L5 with epidural component; initially diagnosed as fibrosarcoma	NR	SMA+, desmin focal + ESOS confirmed on histopathology	Preoperative RT + *en bloc* R0 resection+partial vertebrectomy	NR (poor prognosis noted for spinal sarcomas)	First report of RP ESOS with epidural/spinal invasion; multidisciplinary management essential
Hu et al. (2014) (20)	52M	5 × 6 → 10 × 10 (at 4 mo)	SPECT/CT: large calcifications, intense Tc-MDP uptake; hepatic capsular, renal fascial, peritoneal metastases	NR	Vimentin+, S100+ CK−, CD117−, CD34−, EMA− Melanoma marker−, Bcl-2−, CD99−	Unresectable; second-look surgery at 4 mo; 2 courses palliative chemo	DOD ∼1 yr	First SPECT/CT documentation of RP ESOS; aggressive peritoneal dissemination; see Liu et al. (21) for clinical details
Liu et al. (2014) (21)	52M	5 × 6 → 10 × 10 → 25 × 30 (9 mo)	Gastric wall implantation metastasis identified; insidious progression	NR	Vimentin+, S100+ CK−, CD117−, CD34−, EMA− Melanoma marker−, Bcl-2−, CD99− ALP: normal → 199 U/L	Surgery × 3 + 2 courses chemo (Endostar+cisplatin+epirubicin)	DOD 1 yr	Gastric wall implantation metastasis (1.1 × 1.2 cm) from peritoneal shedding; rare metastatic pattern; see Hu et al. (20) for SPECT/CT
Ratnagiri et al. (2012) (22)	46M	NR	CT: tumor at lower pole of left kidney, infiltrating descending colon; presented as sub-acute intestinal obstruction	NR	No IHC; diagnosis by morphology: malignant spindle cells, pleomorphic nuclei, atypical mitoses, abundant malignant osteoid (osteoblastic variant)	R0 resection (left radical nephrectomy+colon resection) + adriamycin-ifosfamide ×6	DOD 6 mo (bilateral pulmonary metastases)	Osteoblastic variant; 5-yr survival 12–25%; chemoresistance noted
Zhang et al. (2010) (15)	74F	16 × 15 × 10	CT + angiography: large heterogeneous RP mass, massive patchy calcification/ossification, peripheral enhancement; hypervascular (left phrenic+renal arteries)	NR	NR	TACE (doxorubicin+oxaliplatin+lipiodol); symptom palliation without size reduction	Died 18 mo after TACE (acute MI)	First report of angiographic features and TACE for RP ESOS; ALP 124 U/L; invaded left kidney+pancreatic tail
Arai et al. (2010) (23)	62F	14 × 11 × 8 → 31 × 30 (recurrence)	CT: large RP mass with diffuse calcification	NR	No IHC reported; diagnosis by morphology: woven bone trabeculae, reverse zoning, atypical cartilage, osteoclastic giant cells; no fatty differentiation	Resection only; no adjuvant therapy	Recurrence at 31 mo (anaplastic spindle cell sarcoma, ∼50/10 HPF); NED after re-resection	Well-differentiated variant; dedifferentiated at recurrence; CT surveillance q6 mo
Schneider et al. (2008) (24)	57M	NR	CT: RP mass involving infrarenal aorta and IVC	NR	NR	En bloc resection+aortic graft (14 mm polyester) + IVC graft (16 mm ePTFE) + right nephrectomy	DOD 105 d post-op (pulmonary metastases); grafts patent at 12 d	First combined aorta + IVC replacement for RP ESOS; prior abdominal wall/spermatic cord ESOS
Secil et al. (2005) (17)	74F	NR	CT/MRI: RP soft-tissue mass, central amorphous calcifications; invaded psoas, quadratus lumborum, diaphragmatic crus	NR	NR	Unresectable; partial excision only	NR (no follow-up)	Radiology teaching case (Case 89); ALP 1420 U/L (markedly elevated); unresectable at surgery
Hamdan et al. (2005) (25)	58M	6.5 × 6.0 × 4.5	CT: left RP mass with calcified/ossified areas; FDG-PET SUVmax >4.5; 99mTc-oxidronate bone scan positive; 2nd smaller mass at splenic lower pole	NR	No IHC reported; biopsy: osteoid network surrounding large atypical cells with necrosis	Unresectable (major vascular invasion); palliative chemo	NR (palliative)	First complete FDG-PET+bone scan characterization of RP ESOS; dual metabolic/osteoblastic phenotype
van Rijswijk et al. (2001) (16)	68M	19	CT: right RP large heterogeneous mass, medial mineralization, lateral necrosis/hemorrhage; MRI: pseudocapsule, T1 intermediate, T2 hyperintense (necrosis); no skeletal primary	NR	Vimentin + (diffuse) S100 focal + (chondroid areas) Neurofilament−, p53−	Complete surgical resection; no adjuvant therapy	No long-term follow-up	Elderly male (68 yr); mild ALP elevation (140 U/L); pseudocapsule on MRI
Wesseling et al. (1990) (26)	Male (adult)	NR	NR	NR	NR	NR	NR	Earliest radiologic description of RP ESOS (letter); limited clinical data

ESOS, extraskeletal osteosarcoma; RP, retroperitoneal; NED, no evidence of disease; NR, not reported; DOD, died of disease; F, female; M, male; mo, months; yr, years; IHC, immunohistochemistry; FISH, fluorescence *in situ* hybridization; TACE, transarterial chemoembolization; G-CSF, granulocyte colony-stimulating factor; MAP, methotrexate + doxorubicin + cisplatin; SPECT/CT, single photon emission CT; FDG-PET, fluorodeoxyglucose PET; ALP, alkaline phosphatase; HPF, high-power field; SUVmax, maximum standardized uptake value; RT, radiotherapy; SD, stable disease; MI, myocardial infarction; ND, not done.

The diagnosis was approached in a stepwise fashion. CT findings offered an important diagnostic hint: the mass contained calcifications but completely lacked macroscopic fat. That observation alone does not exclude a tiny well-differentiated component; it merely shifts the probability. The definitive answer came from the surgical specimen. The entire tumor was examined at 1 cm intervals and no well-differentiated liposarcoma component was found. Histology showed high-grade sarcoma with lace-like osteoid, a pattern shared by both dedifferentiated liposarcoma and extraskeletal osteosarcoma. Morphology alone cannot distinguish them; molecular testing can.

MDM2 amplification is the genetic hallmark of dedifferentiated liposarcoma (12). The FISH result was negative. CDK4 was tested by FISH as well, because CDK4 co-amplification provides additional assurance, and was also negative. Negative CDK4 immunostaining further supports the absence of 12q13–15 amplification (13). Taken together with the exhaustive search for a well-differentiated component, the evidence against that diagnosis is strong. For future cases, a tissue core should be reserved for molecular studies at the time of initial sampling, particularly in large, heterogeneous tumors where subsequent immunohistochemistry can consume the block. One limitation of the molecular work-up is that EWSR1 rearrangement testing was not performed; because CD99 is not entirely specific, extraskeletal Ewing sarcoma was therefore not excluded at the molecular level, a distinction that is clinically relevant because Ewing sarcoma is generally more chemosensitive than extraskeletal osteosarcoma and would be managed with a different chemotherapy regimen. Nevertheless, the abundant lace-like osteoid production throughout a pleomorphic high-grade sarcoma is a defining feature of extraskeletal osteosarcoma and is not a feature of Ewing sarcoma.

This case also carries a warning about the decision to bypass biopsy. Biopsy was omitted in the face of rapidly progressive distension and a clearly resectable mass, but NCCN and ESMO guidelines endorse biopsy for virtually all retroperitoneal tumors (6). A preoperative tissue diagnosis would have permitted neoadjuvant therapy if dedifferentiated liposarcoma had been identified, or could have altered the surgical approach had a different diagnosis (for example, lymphoma or Ewing sarcoma) been found. This choice was a conscious exception, not a justification; clinicians should adhere to guideline-based biopsy protocols.

The present case carries several distinctive features that extend the current understanding of retroperitoneal extraskeletal osteosarcoma. First, the patient was a 33-year-old woman without prior radiation or known predisposition syndrome, reinforcing that this diagnosis can arise in young adults and is not restricted to older populations. Among the 14 previously reported cases, the median age was 56 years and only 2 other patients were under 40 [Table 1: Jaipuria et al. (14), 38 years]. Second, the imaging findings were notably subtle: only punctate and amorphous calcifications were present, without the dense, organized ossification described in several prior reports [Table 1: Zhang et al. (15), van Rijswijk et al. (16), and Secil et al. (17) reported tumors with massive or organized calcification or ossification]. This relative paucity of mineralized matrix on CT makes the tumor easier to misclassify as a soft-tissue sarcoma of uncertain lineage. Third, the systematic stepwise diagnostic approach (imaging, exhaustive gross sampling, histology, immunohistochemistry, and confirmatory FISH for both MDM2 and CDK4) provides a template for distinguishing extraskeletal osteosarcoma from dedifferentiated liposarcoma. None of the previously reported cases underwent systematic dual FISH testing for both MDM2 and CDK4 (Table 1). The key educational message is that a retroperitoneal osteoid-producing sarcoma without a well-differentiated liposarcoma component should not be presumed to be dedifferentiated liposarcoma without molecular exclusion of 12q13–15 amplification. A systematic review of the 14 previously published cases is provided in Table 1.

Whether adjuvant chemotherapy helps in extraskeletal osteosarcoma remains unsettled. The evidence supporting chemotherapy in skeletal osteosarcoma is robust (10); for the extraskeletal form it is much weaker and largely retrospective. The Japanese registry study provides a signal of potential benefit but is prone to bias (11). The MAP regimen was used because of its proven efficacy in high-risk skeletal osteosarcoma; the patient was informed fully about the uncertain magnitude of benefit and the potential toxicities. Her 25-month disease-free interval is an early observation and offers no guarantee against late failure. Given that 5-year survival in extraskeletal osteosarcoma hovers around 30% based on older series (3), and the outcomes in Table 1 reinforce this unfavorable prognosis in the retroperitoneal subset, surveillance is planned for at least a decade.

## Conclusion

This case illustrates that retroperitoneal extraskeletal osteosarcoma can present with subtle imaging features that overlap substantially with dedifferentiated liposarcoma. When a retroperitoneal sarcoma produces osteoid and lacks a well-differentiated liposarcoma component on imaging, gross examination, and microscopy, MDM2 amplification testing, and ideally CDK4 co-testing, is essential to exclude dedifferentiated liposarcoma. In this patient, negative FISH results for both MDM2 and CDK4, together with exhaustive negative sampling for a well-differentiated component, established the diagnosis of primary extraskeletal osteosarcoma and directed her toward osteosarcoma-specific adjuvant therapy. The 25-month disease-free interval is an early observation that does not preclude late recurrence; long-term surveillance remains necessary. Preoperative biopsy remains the standard of care and should not be bypassed without compelling multidisciplinary justification.

## Data Availability

The original contributions presented in the study are included in the article/Supplementary Material, further inquiries can be directed to the corresponding authors.
